# Variation of T_2_ relaxation times in pediatric brain tumors and their effect on metabolite quantification

**DOI:** 10.1002/jmri.26054

**Published:** 2018-04-26

**Authors:** Dominic Carlin, Ben Babourina‐Brooks, Nigel P. Davies, Martin Wilson, Andrew C. Peet

**Affiliations:** ^1^ Institute of Cancer and Genomic Sciences University of Birmingham Birmingham West Midlands UK; ^2^ Birmingham Children's Hospital NHS Foundation Trust Birmingham West Midlands UK; ^3^ Imaging and Medical Physics University Hospitals Birmingham NHS Foundation Trust Birmingham West Midlands UK; ^4^ Birmingham University Imaging Centre (BUIC), School of Psychology University of Birmingham West Midlands UK

**Keywords:** T_2_ relaxation, pediatric brain tumors, MRS quantification, MRS

## Abstract

**Background:**

Metabolite concentrations are fundamental biomarkers of disease and prognosis. Magnetic resonance spectroscopy (MRS) is a noninvasive method for measuring metabolite concentrations; however, quantitation is affected by T_2_ relaxation.

**Purpose:**

To estimate T_2_ relaxation times in pediatric brain tumors and assess how variation in T_2_ relaxation affects metabolite quantification.

**Study Type:**

Retrospective.

**Population:**

Twenty‐seven pediatric brain tumor patients (*n* = 17 pilocytic astrocytoma and *n* = 10 medulloblastoma) and 24 age‐matched normal controls.

**Field Strength/Sequence:**

Short‐ (30 msec) and long‐echo (135 msec) single‐voxel MRS acquired at 1.5T.

**Assessment:**

T_2_ relaxation times were estimated by fitting signal amplitudes at two echo times to a monoexponential decay function and were used to correct metabolite concentration estimates for relaxation effects.

**Statistical Tests:**

One‐way analysis of variance (ANOVA) on ranks were used to analyze the mean T_2_ relaxation times and metabolite concentrations for each tissue group and paired Mann–Whitney *U*‐tests were performed.

**Results:**

The mean T_2_ relaxation of water was measured as 181 msec, 123 msec, 90 msec, and 86 msec in pilocytic astrocytomas, medulloblastomas, basal ganglia, and white matter, respectively. The T_2_ of water was significantly longer in both tumor groups than normal brain (*P* < 0.001) and in pilocytic astrocytomas compared with medulloblastomas (*P* < 0.01). The choline T_2_ relaxation time was significantly longer in medulloblastomas compared with pilocytic astrocytomas (*P* < 0.05), while the T_2_ relaxation time of NAA was significantly shorter in pilocytic astrocytomas compared with normal brain (*P* < 0.001). Overall, the metabolite concentrations were underestimated by ∼22% when default T_2_ values were used compared with case‐specific T_2_ values at short echo time. The difference was reduced to 4% when individually measured water T_2_s were used.

**Data Conclusion:**

Differences exist in water and metabolite T_2_ relaxation times for pediatric brain tumors, which lead to significant underestimation of metabolite concentrations when using default water T_2_ relaxation times.

**Level of Evidence:** 3

**Technical Efficacy:** Stage 2

J. Magn. Reson. Imaging 2019;49:195–203.

BRAIN TUMORS are the most common solid tumors in children and a significant cause of morbidity and mortality. ^1^H magnetic resonance spectroscopy (MRS) provides a noninvasive means of profiling the chemical composition of brain tumors, providing prognostic and diagnostic biomarkers that can be used for tumor classification^1^, ^2^ and for monitoring treatment response.^3^ As quantitative metabolite biomarkers start to be proposed for clinical decision‐making in individual patients,^4^, ^5^ accurate measurement becomes of increasing importance.

MRS is typically implemented clinically by adhering to an agreed protocol and comparing the results to those obtained using the same protocol. This strategy is known to be reproducible when a single scanner is involved^6^ and a reasonable comparison can also be made between different scanners and centers providing the protocol is adhered to.^2^ While metabolite levels can be reported as ratios, quantification is typically performed by fitting to a set of metabolite basis functions and concentrations are calculated with reference to an unsuppressed water signal.^7^

Popular analysis packages, LCModel^8^ and TARQUIN,^9^ are often used for metabolite quantification, assuming T_2_ relaxation times typical of normal brain in their calculations. However, relaxation times are sensitive to microenvironment and have been shown to change with pathology.^10^, ^11^, ^12^, ^13^ In the previous major studies of brain tumors using MRS, further correction for the differences in T_2_ relaxation between brain tumors and healthy brain has not been performed and the effects of T_2_ relaxation variation have been assumed to be small at short echo time (TE).^1^, ^2^, ^14^

The use of longer TEs for metabolite quantification has been proposed recently^12^, ^15^, ^16^, ^17^; however, T_2_ relaxation times are known to have a significant effect on concentration determination at long TEs^18^ and correction for T_2_ relaxation has been shown to have a significant effect on metabolite ratios at 3T.^13^ Accurate water and metabolite T_2_ relaxation times are therefore likely to be required for reliable metabolite quantification. With the emergence of various acquisition protocols, it is becoming increasingly important to explore the effects of relaxation on quantification at various TEs.

In a recent multicenter study of pediatric brain tumors,^2^ reporting concentrations measured at both short‐ and long‐TE, a large difference was seen between the concentrations measured at the two echo times. Although a recent study has examined the effect of using long‐TEs for quantification using LCModel,^18^ the influence of T_2_ relaxation times on brain tumor quantification at short‐TE was not formally assessed.

While previous studies of adult brain tumors have shown significant differences in both water and metabolite T_2_ relaxation times,^10^, ^11^, ^12^, ^13^ T_2_ relaxation in childhood brain tumors has not been extensively studied to date. Investigation of relaxation times in the pediatric population is of particular importance, as metabolite T_2_ relaxation times in normal brain have been shown to change with age.^19^ In addition, for brain tumors, specific studies in children are required since the tumors are histologically and biologically different from their adult counterparts.^20^, ^21^ Pilocytic astrocytomas and medulloblastomas are particularly common in children but are rare in adults.^22^, ^23^

Measuring T_2_ formally is challenging since acquisition protocols require multiple echo times and this leads to long acquisition times. However, protocols using two echo times have been implemented clinically and offer the potential to estimate the T_2_ values of metabolites and water while keeping acquisition times within reasonable timeframes.^2^, ^10^ This issue is particularly pertinent to the study of children, where long scans are poorly tolerated.

In this study, metabolite and water T_2_ relaxation times in apparently normal brain and childhood brain tumors were retrospectively calculated from data collected at both short‐ and long‐TE with the aim of establishing how the relaxation properties of major metabolites vary and the effect this has on metabolite quantification.

## Materials and Methods

### Patients

Two cohorts were retrospectively selected from patients where single‐voxel MRS had been performed prior to treatment between September 2006 and July 2011. The first cohort consisted of 27 children with brain tumors. This tumor group was comprised of 10 medulloblastomas (six male and four female, mean age 6.1 years) and 17 pilocytic astrocytomas (nine male and eight female, mean age 7.4 years).

Comparison was made with a second cohort consisting of 24 age‐matched children (18 male and 6 female, mean age 6.4 years). These children had MRI and MRS as part of an investigation for a suspected metabolic disorder. Metabolic disorders were subsequently ruled out for these children and all children had normal‐appearing MRI and MRS. All patients were under 16 years of age and contemporaneous informed parental consent and research ethics approval was obtained that covered future analyses.

### MRS Acquisition

MRS was acquired using a Siemens Symphony Magnetom NUM4 1.5T scanner (Siemens Healthcare, Erlangen, Germany) following conventional imaging. The standard imaging set of T_1_‐weighted, T_2_‐weighted, and T_1_‐weighted images postcontrast administration was used to delineate the tumor margins. Cubic voxels of side length 1.5 cm or 2 cm were placed entirely within the solid component of the tumor, avoiding any cyst or necrosis, and point‐resolved spectroscopy (PRESS) was performed. Cubic voxels of side length 2 cm were placed in the basal ganglia and parietal white matter in the cohort with normal‐appearing MRI and MRS. Water‐suppressed data were acquired with 128 repetitions from the larger voxels and 256 repetitions from the smaller ones. For all MRS acquisitions, a relaxation time (TR) time of 1500 msec was used and data were acquired at both short (30 msec) and long (135 msec) TE from the same voxel. Water‐unsuppressed MRS data were also acquired with four repetitions as a concentration reference at both TEs.

### Processing and Analysis

Raw spectroscopy data were automatically processed using TARQUIN v. 4.3.8.^9^ TARQUIN models experimental data as a linear combination of simulated basis signals. To extract metabolite concentrations, the fitted signal amplitudes â are scaled by two factors: W_att_ and W_conc_.

The W_att_ parameter accounts for the reduction of the water signal relative to metabolite signals due to differences in T_2_ relaxation at a given TE and is defined as W_att_ = [exp(–TE/T_2water_) / exp(–TE/T_2metabolite_)]. This parameter is used to adjust the metabolite concentrations to be independent of TE and will give the correct value if T_2water_ and T_2metabolite_ are known accurately. W_conc_ denotes the assumed water concentration for a given tissue type and is used to scale â to the amplitude of the unsuppressed water signal. W_conc_ and W_att_ are assumed as 35,880 mM and 0.7, respectively, by TARQUIN as the default.

Tumor spectra were referenced to the total choline signal to account for frequency drift, while normal brain spectra were referenced to a combination of total choline‐creatine‐NAA‐lipids. The following metabolite, lipid, and macromolecule signals were included in the basis set: alanine (Ala), aspartate (Asp), γ‐aminobutyric acid (GABA), glycerophosphocholine (GPC), glucose (Glc), glutamine (Gln), glutathione (Glth), glutamate (Glu), glycine (Gly), myo‐Inositol (mI), lactate (Lac), N‐acetylaspartate (NAA), N‐acetylaspartylglutamate (NAAG), phosphocholine (PCh), phosphocreatine (PCr), scyllo‐Inositol (Scy), taurine (Tau), lipids at 0.9, 1.3 (a+b), and 2.0 ppm and macromolecules at 0.9, 1.2, 1.4, 1.7, and 2.0 ppm. Due to significant spectral overlap at 1.5T, the following metabolites were combined in the subsequent analysis: Gln + Glu = Glx; NAA + NAAG = tNAA; Cr + PCr = tCr and GPC + PCh = tCho.

#### T_2_ Measurement

T_2_ relaxation times were estimated by fitting TARQUIN estimates of signal amplitude â^9^ for each metabolite from the two echo times to a monoexponential function. Two expert spectroscopists (D.C. and B.B.B., 4 years of experience each) assessed the presence of tNAA, tCho, and tCr in short‐ and long‐TE MRS independently. T_2_ relaxation times were estimated for the cases where both assessors agreed that the metabolites could be identified at both TEs from visual inspection.

#### Concentration Calculation

To assess the importance of accurate T_2_ relaxation times for metabolite quantification, we compared metabolite concentrations corrected using combinations of the measured T_2_ values and using literature T_2_ values. The following terms are used for the various methods of obtaining the T_2_ values. Tissue type refers to pilocytic astrocytoma, medulloblastoma, or normal brain.

Individual water (IW) correction uses the T_2_ relaxation time of water measured as part of the MRS acquisition for that case.

Average metabolite (AM) correction uses the average T_2_ relaxation time for the metabolites obtained from the cases in the study with the same tissue type for tNAA, tCho, and Cr and a metabolite T_2_ of 400 msec for all other metabolites.

For literature T_2_ correction, water T_2_s were assumed to be 86 msec for normal brain.^24^ Metabolite T_2_s of 368.8, 205.3, and 265.4 msec were used for tNAA, tCr, and tCho, respectively, in healthy brain.^25^ As T_2_ relaxation has been relatively unexplored in childhood pilocytic astrocytoma and medulloblastoma, literature correction for our brain tumor cohorts used published T_2_ values for adult gliomas.^11^ The water T_2_ relaxation times were taken to be 174.5 msec in both brain tumor types. The T_2_ values for tNAA, tCr, and tCr were 227.5, 196.3, and 275.3 msec, respectively.

The term default is used for concentrations estimated using TARQUIN's default W_att_ value of 0.7. This value is based on TE = 30 msec data collected in adults at 1.5T and assumes T_2_ relaxation times typical of white matter.

Metabolite concentrations quantified at short‐TE were corrected for relaxation effects by calculating case‐specific W_att_ values using AM and IW T_2_ values, for which we use the shorthand notation (AM, IW). These values are assumed to be the best estimates of the metabolite values and all other methods are compared against these with the root mean square (rms) differences being reported.

### Quality Control

MRS data were required to have a water linewidth (full‐width‐at‐half maximum, FWHM) <15 Hz and signal‐to‐noise ratio (SNR) ≥4. Spectra were reviewed for quality by two expert spectroscopists. Short‐ and long‐TE spectra were assessed to identify metabolites present at both echo times for inclusion in the T_2_ analysis.

### Statistics

One‐way analysis of variance (ANOVA) on ranks tests were used to analyze the T_2_ relaxation times and metabolite concentrations for the four tissue groups and paired Mann–Whitney *U*‐tests were performed. Statistical significance was declared for *P* < 0.05.

## Results

**Figure 1:**
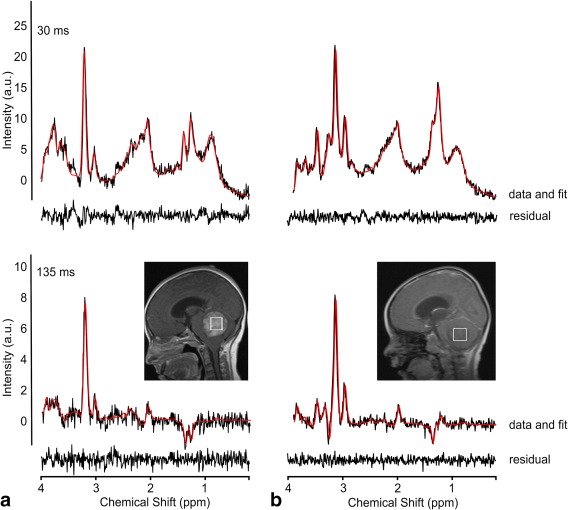
Example spectra from **(a)** pilocytic astrocytoma astrocytoma and **(b)** medulloblastoma at short‐ and long‐TE with TARQUIN fits (red) and fit residuals shown beneath the spectra.

**Figure 2:**
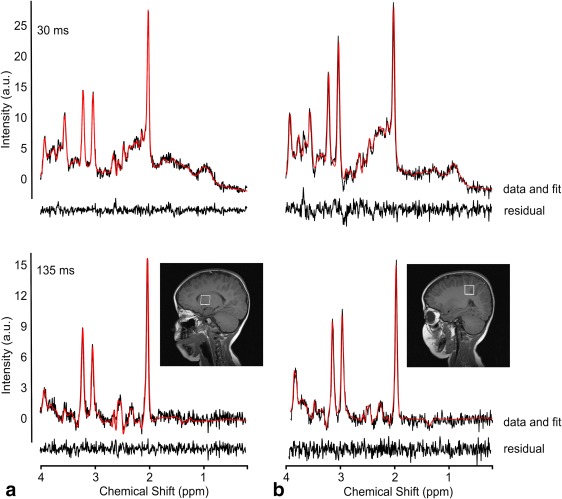
Example spectra from **(a)** basal ganglia and **(b)** white matter at short‐ and long‐TE with TARQUIN fits (red) and fit residuals shown beneath the spectra.

After quality control, 11 pilocytic astrocytoma, 10 medulloblastoma, 15 white matter, and 16 basal ganglia spectra were used in the analysis. A total of 45 tNAA (10 pilocytic astrocytomas, 5 medulloblastoma, 14 white matter, and 16 basal ganglia), 43 tCr (8 pilocytic astrocytomas, 9 medulloblastoma, 10 white matter, and 16 basal ganglia), and 49 tCho (11 pilocytic astrocytomas, 9 medulloblastoma, 15 white matter, and 14 basal ganglia) were included in the T_2_ analysis. Figure 1 shows examples pilocytic astrocytoma and medulloblastoma spectra. Figure 2 shows example basal ganglia and white matter spectra.

### T_2_ Results

**Figure 3:**
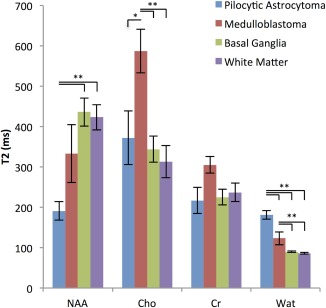
Mean (standard error) T_2_s of tNAA, tCho, tCr, and water for pilocytic astrocytomas, medulloblastomas, basal ganglia, and normal white matter. *P < 0.05, **P < 0.001.

T_2_ values for metabolites and water are represented in Fig. 3, with the values given in Table 1. tCho was significantly longer in medulloblastomas compared with pilocytic astrocytoma (*P* = 0.04) and compared with white matter (*P* < 0.001) and basal ganglia (*P* < 0.001). The T_2_ relaxation time of tNAA was significantly shorter in pilocytic astrocytomas compared with white matter (*P* < 0.001) and basal ganglia (*P* < 0.001).

**Table 1:** Estimated Metabolite T2 Relaxation Times (msec) in Pilocytic Astrocytoma, Medulloblastoma, Basal Ganglia (BG), and White Matter (WM)

	T2 relaxation time (msec)							
	Pilocytic astrocytomas		Medulloblastomas		BG		WM	
	Mean SD	*n*	Mean SD	*n*	Mean SD	*n*	Mean D	*n*
tNAA	191 ± 56	10	333 ± 124	5	436 ± 140	16	423 ± 113	14
tCho	372 ± 176	11	587 ± 143	9	344 ± 122	14	313 ± 154	15
tCr	217 ± 65	8	305 ± 51	9	225 ± 78	16	237 ± 72	10
Water	181 ± 35	11	123 ± 45	10	90 ± 9	16	86 ± 8	15

The T_2_ TRs of tissue water in pilocytic astrocytomas 181 ± 35 msec and medulloblastomas 123 ± 45 msec were found to be significantly longer than in basal ganglia, 90 ± 9 msec, and white matter 86 ± 8 msec (*P* = 10^−6^ in all cases). The T_2_ TR of water was significantly longer in pilocytic astrocytomas than in medulloblastomas (*P* = 0.001).

The mean W_att_ values, calculated using the mean tissue water and metabolite T_2_ TRs, were 0.95, 0.85, and 0.80 for pilocytic astrocytomas, medulloblastomas, and normal brain, respectively, at TE = 30 msec. There was no correlation between T_2_ TRs and age in normal brain for water or any of the metabolites.

### Effect of T_2_ Correction on Metabolite Concentrations

**Figure 4:**
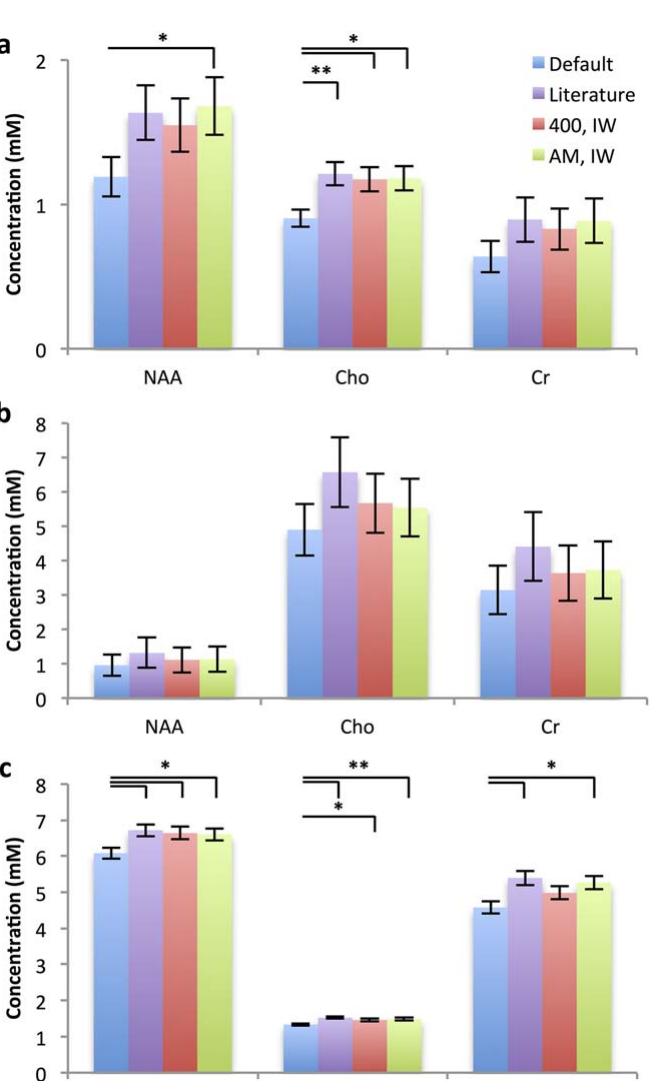
Mean ± standard error metabolite concentrations (mM) in **(a)** pilocytic astrocytoma, **(b)** medulloblastoma, and **(c)** normal brain estimated at short‐TE. Metabolite concentrations are corrected for relaxation effects using either default TARQUIN relaxation correction; literature T_2_ values; a metabolite T_2_ of 400 msec and a patient measured water (IW) T_2_; or the average metabolite (AM) T_2_ for that tissue type and IW T_2_. *P < 0.05, **P < 0.01.

Metabolite concentrations were measured and adjusted using various combinations of T_2_ values: default T_2_ values; literature T_2_ values; a value of 400 msec for metabolite T_2_ and individually measured water T_2_ (IW); average metabolite T_2_ (AM) and IW. A comparison of mean concentrations for tNAA, tCho, and tCr determined using these protocols is shown in Fig. 4.

Concentrations corrected using the default protocol were significantly underestimated compared with those estimated using the AM, IW correction regime (*P* < 0.05). However, concentrations estimated using different metabolite T_2_ values were comparable, providing the same water T_2_ value was used for correction. When averaged over all metabolites and all tissue types, the mean short TE MRS metabolite concentrations calculated using the default T_2_ TRs were significantly underestimated by 22% compared with the best estimate mean concentrations determined using the IW and AM TRs (*P* < 0.001). Metabolite concentrations were underestimated by 4% when a metabolite T_2_ of 400 msec and IW T_2_ were used.

The rms error of metabolite concentrations estimated using (default T_2_) and (400 msec, IW) relaxation times compared with those using (AM, IW) relaxation times are presented in Table 2 for each tissue type. The rms errors observed at short‐TE were significantly smaller than those at long‐TE when observing the patient cohort as a whole (24% vs. 58% for default T_2_s vs. AM, IW, *P* <0.001).

**Table 2:** Root Mean Square Percentage Difference Between Metabolite Concentrations Corrected Using Different Combinations of T2 Relaxation Times (see Materials and Methods) Compared to the Corrected Concentration Using the Patient's Measured T2 Values (AM, IW)

	Pilocytic astrocytoma		
	Default	Literature	400, IW
Short TE singlets	31.1	5.5	6.0
Long TE singlets	46.4	19.8	26.1

	Medulloblastoma		
	Default	Literature	400, IW
Short TE singlets	17.6	16.1	1.9
Long TE singlets	54.4	69.5	9.7

	Normal brain		
	Default	Literature	400, IW
Short TE singlets	13.9	3.8	3.3
Long TE singlets	73.8	16.9	14.8

A, average; I, individual; M, metabolite; W, water.

### Mean Metabolite Concentrations Corrected for T_2_ Relaxation Times for Each Tissue Type

Mean metabolite concentrations for the different tissue types calculated using an IW, AM T_2_ corrected protocol are presented in Table 3. The concentrations of tNAA (*P* < 0.001), tCr (*P* < 0.001), and Glx (*P* < 0.001) were significantly lower in tumors compared with normal brain, while Lac (*P* = 10^−4^) was significantly higher in tumors. The concentration of tCho (*P* < 0.001), Tau (*P* < 0.01), Gly (*P* < 0.01), and tCr (*P* < 0.05) were significantly higher in medulloblastomas compared with pilocytic astrocytomas.

**Table 3:** Estimated Mean ± SD Metabolite Concentrations (mM) of Pilocytic Astrocytomas, Medulloblastomas, and Normal Brain, Corrected for Individually Estimated Water and Average Metabolite T2 Relaxation Times

Concentration (mM)						
		Pilocytic astrocytomas	Medulloblastomas		Normal	
	ANOVA all groups (*P* value)	Mean SD	Mean SD	Pilocytic astrocytoma Vs. medulloblastoma (*P* value)	Mean SD	Normal vs. all tumors (*P* value)
tNAA	10^−6^	1.62 ± 0.66	1.25 ± 1.35	0.36	7.44 ± 4.45	10^−7^
tCho	10^−9^	1.20 ± 0.40	5.50 ± 2.24	< 0.001	1.62 ± 0.64	< 0.01
tCr	10^−7^	0.86 ± 0.61	3.74 ± 2.28	< 0.01	5.69 ± 2.10	10^−6^
Lac	10^−7^	1.35 ± 0.59	4.03 ± 2.47	0.01	0.32 ± 0.97	10^−6^
Ala	0.06	0.29 ± 0.36	0.30 ± 0.46	0.73	0.12 ± 0.17	0.02
mI	0.15	1.12 ± 1.14	3.08 ± 3.13	0.17	3.48 ± 3.72	0.09
Tau	10^−7^	0.65 ± 0.47	4.39 ± 2.19	< 0.001	0.98 ± 1.23	< 0.01
Gly	10^−10^	0.33 ± 0.54	4.15 ± 1.87	< 0.001	0.42 ± 0.62	< 0.001
Scy	10^−5^	0.00 ± 0.00	0.65 ± 0.51	< 0.01	0.19 ± 0.14	0.25
Glx	< 0.01	5.22 ± 2.59	5.20 ± 2.75	0.95	7.75 ± 3.95	< 0.001

## Discussion

In this study, T_2_ relaxation times of metabolites and water were estimated in childhood brain tumors and metabolite concentrations corrected for relaxation effects are reported. The importance of T_2_ relaxation times for quantification was also assessed.

In previous major studies of metabolite concentrations in brain tumors,^1^, ^2^, ^14^, ^26^ no correction for the differences in T_2_ relaxation times of brain tumors and healthy brain was performed. A water attenuation factor of 0.7 is applied to the data by default in LCModel and TARQUIN. This value is calculated using data collected at 1.5T in healthy adult brain assuming a TE of 30 msec and is not suitable for quantification of long‐TE data. In the current study, concentrations measured without further correction for the differences in relaxation were typically underestimated by ∼22% at short‐TE.

While there are differences between the concentrations corrected for water and metabolite T_2_ versus those corrected using the default T_2_ values, the main features in the metabolite profiles reported for medulloblastomas and pilocytic astrocytomas in children have been substantiated.^1^, ^2^, ^14^ While previous studies have assessed the influence of T_2_ relaxation on metabolite ratios^13^ and quantification at long‐TE,^18^ this study assessed the variation in relaxation time in pediatric brain tumors and normal brain and the effect on metabolite quantification. The relative importance of water and metabolite T_2_ relaxation times was also assessed. The T_2_ relaxation time of tissue water was found to have a greater effect on concentration measurements than the T_2_ relaxation time of metabolites. An additional multi‐TE acquisition to measure the T_2_ of water can be implemented with a scan time of less than a minute, and is recommended to improve the accuracy of metabolite quantification. This may be particularly pertinent if using quantitative MRS to assess treatment response, as tumor water T_2_ is known to reduce following treatment.^27^, ^28^, ^29^ However, if water T_2_ values for individual cases are not available, then the mean values for the relevant tissue type would be preferable to default values.

At long‐TE, the rms percentage differences from concentrations corrected for both IW and AM T_2_ relaxation were larger than at short‐TE, suggesting that accurate metabolite T_2_ values are of more importance at long echo times than at short. This is as expected, since signal losses due to T_2_ relaxation effects increase with echo time and inaccurate T_2_ estimation will lead to greater errors at longer echo times. Since we detected no significant differences in the T_2_ measurements between tissue types for the majority of metabolites, T_2_ values determined from normal brain could reasonably be used in determining concentrations for tumors. More accurate values are likely to be obtained if metabolite‐specific T_2_s are used, although this is somewhat more challenging to implement.

A number of studies have reported T_2_ relaxation times of adult brain tumors at 1.5^10^, ^11^ and 3 T^13^, ^30^; however, relaxation times in pediatric brain tumors have been relatively unexplored. Consistent with observations of prolonged water T_2_ in adult brain tumors, the T_2_ relaxation time of water was found to be significantly longer in tumors than normal brain and in pilocytic astrocytomas compared with medulloblastomas. The long water T_2_ in brain tumors is consistent with the high signal seen on T_2_‐weighted imaging compared with gray matter and corresponds to the high water content, especially in pilocytic astrocytomas. For absolute metabolite quantification, differences in water content should be accounted for. No correction was made for variations of water tissue content, in keeping with previous studies of brain tumors, and it is not known what error this will introduce on concentration measurements. However, voxels were placed entirely within the solid component of the tumor to exclude all cystic components. Tissue water content is rarely determined in clinical studies and its effect should be the subject of a future study.

The T_2_ of tCho was found to be significantly longer in medulloblastomas compared with pilocytic astrocytomas. The reason for the long T_2_ of tCho in medulloblastoma is uncertain. However, the resonance at 3.20 ppm is composed mainly of PCh in medulloblastomas and GPC in gliomas.^31^, ^32^ A longer PCh T_2_ would be consistent with its lower molecular weight relative to GPC. The significantly lower T_2_ of tNAA in pilocytic astrocytomas compared with normal brain would be consistent with some of the signal around 2 ppm being from a macromolecular component, although it is not sufficiently low for this to explain all the signal.^33^

A limitation of this retrospective study is that only two TEs were used to evaluate T_2_ relaxation times. The relatively short range of TE values used and the bias due to exclusion of cases where the signal could not be accurately fitted at the longer echo time may have led to an overestimation of metabolite T_2_ values.^34^ Lac could not be reliably quantified at short‐TE and quantification of lipids and macromolecules was not possible at long‐TE. For optimal evaluation of the relaxation times of coupled metabolites, appropriate TE values should be determined following evaluation of the J‐evolution of metabolite signals, as overlap of chemically inequivalent species will have an effect on the apparent T_2_ of MRS peaks.^35^ However, the optimal TE values vary between metabolites and using additional TEs is prohibitively time‐consuming for routine tumor evaluation in a clinical environment. If a dual echo time acquisition is to be used, we do not currently know the optimum pair of values, but a shorter second echo time than used in this study could allow for more cases to be assessed due to the higher SNR at shorter‐TEs. Estimation of water T_2_ values requires a much shorter acquisition due to its high signal intensity and could readily be included as part of the routine protocol.

While we have investigated the effects of T_2_ relaxation on metabolite determination, no attempt was made to correct for T_1_ saturation effects. In clinical studies, a TR of 1500 msec is typically used in pediatric single‐voxel spectroscopy studies at 1.5T.^1^, ^2^, ^14^ This relative consistency should provide comparability of data between studies. A previous study of relaxation effects in adult brain tumors found no significant differences in the T_1_ between metabolites or between tissue types,^10^ implying that metabolite values may be comparable even if a different TR is used.

Our study design has a number of limitations. First, the study was conducted retrospectively and the number of patients included in the analysis was relatively small. Second, we used AM, IW concentration estimates as our standard, as the precise metabolite concentrations are unknown. Although our method will reduce errors introduced by misestimation of metabolite relaxation times, this choice is somewhat arbitrary. Finally, measuring the T_2_ relaxation times postcontrast may have introduced some errors into our T_2_ estimates. Our T_2_ estimates may therefore be underestimated by as much as 16%; however, preclinical evidence suggests that there is little difference in the T_2_ relaxation times at different moments of the contrast washout.^36^

In conclusion, T_2_ relaxation times of water and metabolites vary between tissue types in children. Using a short echo time and correcting for T_2_ effects with the best values available, preferably including case‐specific water T_2_ values, will reduce inaccuracies due to T_2_ variability. T_2_ values themselves are a measure of molecular environment and may provide an additional means of investigating and characterizing tissue.
